# Pupil response to noxious corneal stimulation

**DOI:** 10.1371/journal.pone.0227771

**Published:** 2020-01-17

**Authors:** Emmanuel B. Alabi, Trefford L. Simpson

**Affiliations:** University of Waterloo, School of Optometry and Vision Science, Waterloo, ON, Canada; Save Sight Institute, AUSTRALIA

## Abstract

**Purpose:**

Ocular somatosensory-autonomic reflexes play critical roles in maintaining homeostasis of the eye. The purpose of this study was to investigate the pupil response to nociceptive corneal stimuli.

**Methods:**

A Waterloo-Belmonte pneumatic esthesiometer was used to determine detection thresholds and randomly deliver mechanical and chemical stimuli from levels of detection threshold to twice the threshold in 50% steps to the central cornea of 15 healthy subjects. For each stimulus, imaging of the stimulated/unstimulated eye was performed using two modified/calibrated Logitech c920 digital cameras for 4 seconds each, pre/post stimulus capture. The data were processed with a custom segmentation algorithm to help identify the pupils and pupil diameter was measured using ImageJ software. Pupil dilation response differences between the ipsi- and contralateral eye was analyzed using dependent t-tests. The effect of stimulus intensity, modality and sex of subjects were analyzed using repeated measures.

**Results:**

In mechanical and chemical stimulation experiments, there was no difference in pupil responses between the stimulated eye and the unstimulated eye, (all dependent T-test *p > 0*.*05*). On average, pupil diameter increased from baseline as the corneal stimulus intensity increased. This happened regardless of whether mechanical or chemical stimulation occurred (ANOVA *p* < 0.05). At 200% threshold, pupil diameter was greater than at all stimulus intensities (Tukey HSD, all *p <* 0.05). Based on stimulus intensity, females had greater pupil diameters than males at levels of 150% threshold and 200% threshold (ANOVA *p* < 0.05, all Tukey HSD *p* < 0.05).

**Conclusion:**

This study serves as a basis for the characterization of the local stimulus-response neural circuitry relating nociceptive stimuli to autonomic responses and in combination with our work on completely separate autonomic circuits of bulbar conjunctival vessel dilation and reflex tearing suggests that the monotonic measurements of redness, tearing and pupils provide accurate, separable responses that reflect painful stimulus intensity.

## Introduction

The International Association for the Study of Pain defined pain as, “an unpleasant sensory and emotional experience associated with actual or potential tissue damage, or described in terms of such damage” [1]. The word “pain” has also been used to describe the experiences associated with discomfort and other unpleasant feelings [2,3]. Pain is subjective and by definition it is experienced only when an individual is in a conscious state, yet the perception and modulation of pain induces brain activity that is driven by autonomic processes that operate below the level of consciousness [4–6]. Previous reports have proposed that autonomic nervous system (ANS) responses have a strong relationship to pain perception and as such, may be possible alternatives for the measurement of pain [7,8].

Within the eye, the ANS controls (among others) two antagonistic iris muscles, the sphincter and dilator pupillae to change pupil size. The sphincter pupillae is innervated by parasympathetic fibers and constricts the pupil, and conversely, the dilator pupillae is innervated by sympathetic fibers and dilates the pupil [9]. Accommodation, luminance, attention, and alertness (among others) cause fluctuations in pupil size [10–12]. The relationship between pupil size changes and pain perception has been looked into quantitatively by various researchers and has been termed pupillary reflex dilation [13], pupil dilation response [14], phasic pupil dilation [14,15], reflex pupillary dilation [16], and ciliospinal reflex [17]. Chapman *et al*. [14] delivered intra-cutaneous noxious fingertip stimulation to 20 subjects at four different intensities and observed a pupillary dilation response. The pupillary dilation response began 0.3 s after delivery of the stimulus and peaked at 1.25s. The researchers concluded that there was a consistent pupillary dilation response to painful systemic stimulation in a dose-response manner.

The pain experience for men and women appear to be different [18,19]. Ellermeier and Westphal [20] suggested females had greater pupil dilation responses than males when tonic pressure was applied to the fingers of subjects, and in the same study, females reported greater pain than males while experiencing the same amounts of noxious stimulation.

On the ocular surface, corneal sensitivity has been shown to vary with age [21–23], time of day [24], and menstruation [25,26] (among other factors). However, there is limited research on the effect of gender on corneal sensitivity [23,27]. Acosta *et al*. [23] reported that in comparison to men of similar age, premenopausal women had lower thresholds to both mechanical and chemical corneal stimulation but, there was no difference between the overall corneal sensitivity of males and females.

Corneal nociceptors receive their innervation from the trigeminal ganglion, via the nasociliary branch of the ophthalmic division of the trigeminal nerve [28,29] and respond to chemical, mechanical and thermal stimulation [30–37]. These neurons have been shown to respond to pneumatic stimulation delivered using the esthesiometer used in the experiment we report: Corneal mechanical response is to ocular surface temperature flow, chemical response is to the protons dissolved in the tears when the stimulus column has additional CO2, and cooling is to room temperature flow [30,35,36].

The autonomic responses to painful ocular stimuli are relatively poorly understood. We have shown that tearing occurs after painful corneal stimulation [38], in a threshold-scaled, dose-response manner and, similarly, bulbar conjunctival vaso-dilation (hyperemia) [39] also occurs in a dose-dependent manner. Understanding these local neural circuits are important in-and-of-themselves, but since ocular inflammatory responses are tied to local pain and redness responses, understanding these mechanisms is important in order to systematically and fully characterize the inflammatory response in humans, in for example, in dry eye [40]. In addition, since pupillary responses themselves occur after painful systemic stimulation, we explored how the pupil changed with these local (corneal) stimuli in order to begin to determine if this autonomic system varied similarly to tearing and redness responses.

The purpose of this exploratory study, then, was to determine whether a pupil response exists for nociceptive corneal mechanical and chemical stimuli, and if so, whether the pupil response is intensity specific. In addition, we were interested in exploring whether the stimulation modality, ipsi- and contralateral effects and whether there were differences in the response between sexes.

## Methods

### Subjects

Ethics clearance (ORE # 19252) was obtained from the Office of Research Ethics at the University of Waterloo before the study began. Eligible subjects signed an informed consent document before enrolment in the study.

15 healthy subjects participated in this study. All subjects underwent a comprehensive ocular examination (with 3 months prior to the study), including an ocular surface work up, where a licensed clinician concluded that they were free from any disease or disorder of the ocular system and appendages. There were 8 male and 7 female volunteers ranging in age from 19 to 34. Subjects on any topical or systemic medication were excluded from the study.

### Waterloo computer-controlled Belmonte esthesiometer

The computer-controlled Belmonte esthesiometer has been described before [31,35,41]. Our modified device used for the delivery of mechanical and chemical stimuli to the ocular surface consists of a control box that electronically regulates the mixture of air and carbon dioxide (CO2)_._ The flow rates of air and concentration of CO2 are separately controlled by two digital flow controllers. Within the nozzle assembly is a thermostat to control temperature. A calibrated video camera was used to ensure that the stimulus was orthogonal to, and the nozzle tip was 5mm from the ocular surface.

### Nociceptive stimuli

Mechanical stimuli consisted of a series of air pulses with varying flow rates from 0 to 200 ml/min and chemical stimulation was delivered by increasing the concentration of CO2 in the air. An ascending methods of limits [42] was used to determine mechanical and chemical detection thresholds of the cornea.

The mechanical threshold, which is the lowest air flow rate (with CO2 set at 0%) that the subject could detect, was first determined. The flow-rate steps were set at 10 mL/min, and the mechanical threshold was the average of three readings when the subject first reported the stimulus. For determining the chemical threshold, the flow rate of air was set at half the initially determined mechanical threshold, and CO2 was added to the air in increments of 5% CO2. The chemical threshold was the average of three first reports of stimulus detection.

### Stimulus delivery

The subjects wore in-ear headphones with white noise playing in the background. The stimulus was presented at the corneal apex of the left eye while subjects viewed a fixation target that was 3 meters away. The tip of the esthesiometer was rotated to ensure the stimulus was delivered perpendicular to the corneal surface during stimulus delivery. The temperature of the air was set to 50°C, this decreased to 33.4°C at the ocular surface at room temperature of 23°C. This was calibrated using a custom electronic thermometer positioned 5 mm from the probe tip (which corresponds to the position of the ocular surface in the experiments). The duration of the stimulus was 2 seconds and it was delivered to the ocular surface immediately after a blink. The subject blinked freely between trials. The next stimulus was triggered after the sensation caused by the last stimulus had disappeared completely.

Once the mechanical and chemical thresholds were determined, a sham (no stimulus was delivered but the participant thought a stimulus was being delivered) and three stimuli were then delivered to the subject in random order, in both the mechanical and chemical stimulation experiments–stimuli at 0%, threshold (sham), stimulus at 150% threshold, and stimulus at 200% threshold. The pupil size prior to stimulus delivery (baseline) and after stimulus delivery were compared.

### Data processing and pupil size measurements

Imaging of the stimulated and unstimulated eye was performed using two modified and calibrated Logitech c920 digital cameras (Logitech c920; Logitech International S.A., Newark, CA), for 4 seconds before (pre-stimulus capture) and 4 seconds after the delivery of the stimulus (post-stimulus capture). The data were processed with a custom segmentation algorithm to help identify the pupils. We then measured the pupil diameter (average of horizontal and vertical measures) using ImageJ software (NIH, Bethesda, MD), for the pre/post capture periods.

The percent (%) change in pupil size was calculated in 2 ways. First, the average pupil size for the 4 seconds after “stimulation” in the baseline (no stimulus) trial was used as the reference and then, the maximum pupil size from the fitted function was used. “% change maximum” being (100*(baseline–max) / baseline) was calculated. Second, % change in average pupil diameter after no stimulation was calculated using the mean of baseline and no stimulus average as the baseline, and pupil diameter after 2x threshold stimulus intensity. “% change average” was (100*(mean pupil diameter after stimulation—mean of baseline)/ mean of baseline).

### Analyses

Preliminary results showed large pupil change effect sizes (of approximately 1.5) and sample size calculations showed approximately 6 participants were necessary to test the (paired) mean difference before and after corneal stimulation [43]. However, in order to stratify by the sex predictor variable, we chose a 15-participant sample size with approximately equal males and female subject numbers.

Initial exploratory examination of pupillary dilation after stimulation was done using generalized additive models (GAMs), fitting smooth functions (spline-based) to pupil size against time, with stimulus intensity, stimulus type (mechanical or chemical) and sex as predictor variables. This was done using the MGCV package in R [44,45]. Non-linear mixed effect models were fit to pupil size against time functions, using the SAEMIX package in R[44], and non-linear regression was done in SPSS for Windows, Version 16.0 (Chicago, SPSS Inc.). Differences in mean pupil diameter using stimulus modality, sex and stimulus intensity predictors were analyzed using repeated measures (RM)-ANOVA and Tukey’s Honestly Significant Difference (HSD) tests for post hoc analysis. Pupil size differences between the ipsi- and contralateral eye was analyzed using dependent t-tests. An alpha value of 0.05 or less was assumed to be significant.

## Results

**Fig 1:**
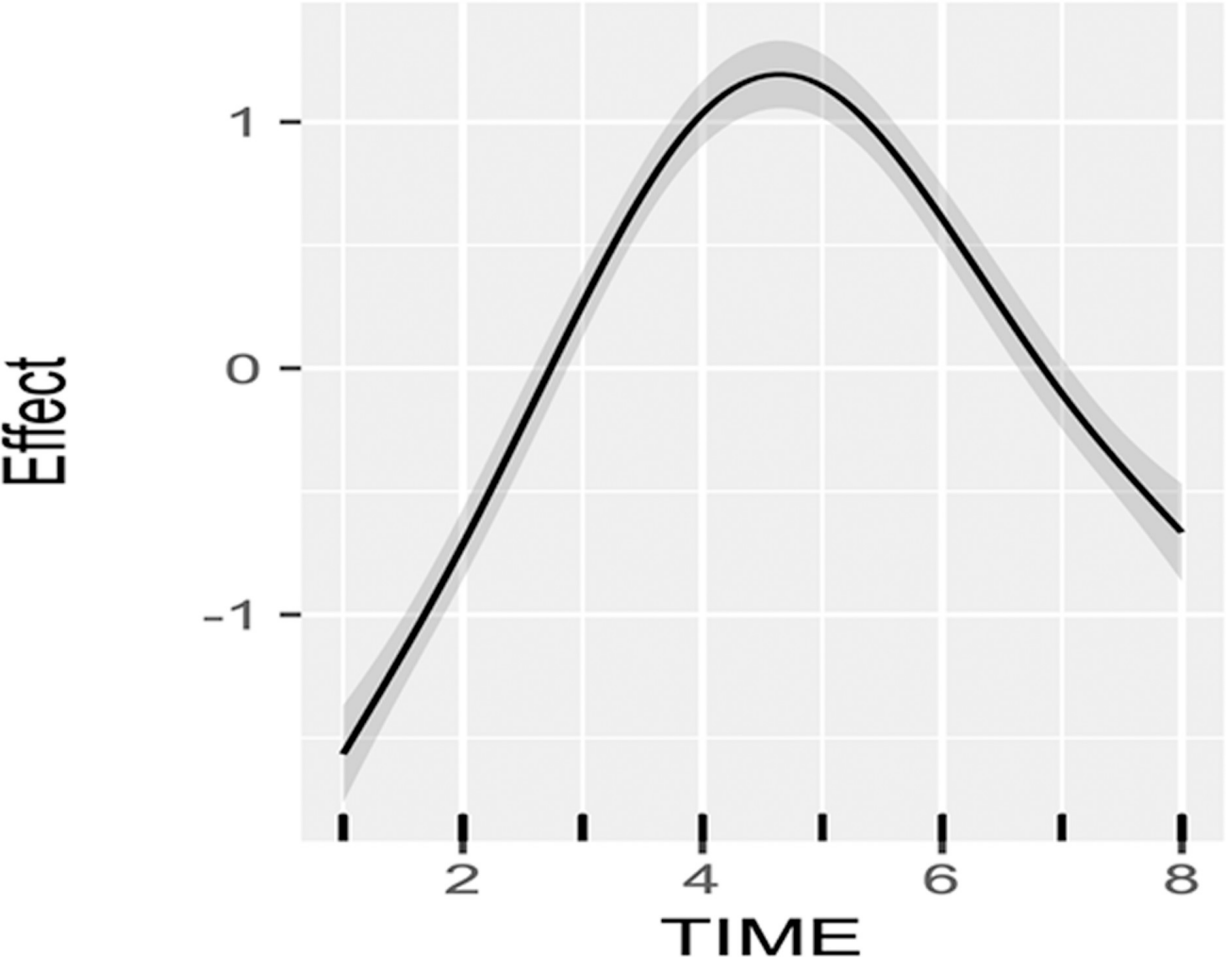
Normalized smooth pupil dilation function (“Effect”, ordinate, normalized mm) versus time (seconds) derived from a generalized additive model with time, sex, and stimulus intensity and type predictor variables. The grey region around the solid line is the approximate 95% confidence interval.

A GAM with a 5 knot spline to smooth the pupil diameter and time function (separately for each stimulus intensity), stimulus type, and sex predictors accounted for approximately 60% of the deviance (R^2^ = 0.6), with significant smooth terms (all p<0.001), and significant main effects of stimulus type, intensity and sex (all p<0.01). This general smoothing function fit to the data, consisting of an initial pupil dilation and an approximate return to near baseline diameter 8 seconds after stimulation is shown in Fig 1.

A similar pupil dilation and constriction function,

$$Pupil\;Size=C+\frac{\left(B*A*Time\right)}{e^{A*Time}}$$

was fit to the data using nonlinear mixed modeling.

After initial exploratory analysis, it was apparent that the confidence intervals of the estimates C commonly included zero, so we fitted a 2-parameter model without C.

**Fig 2:**
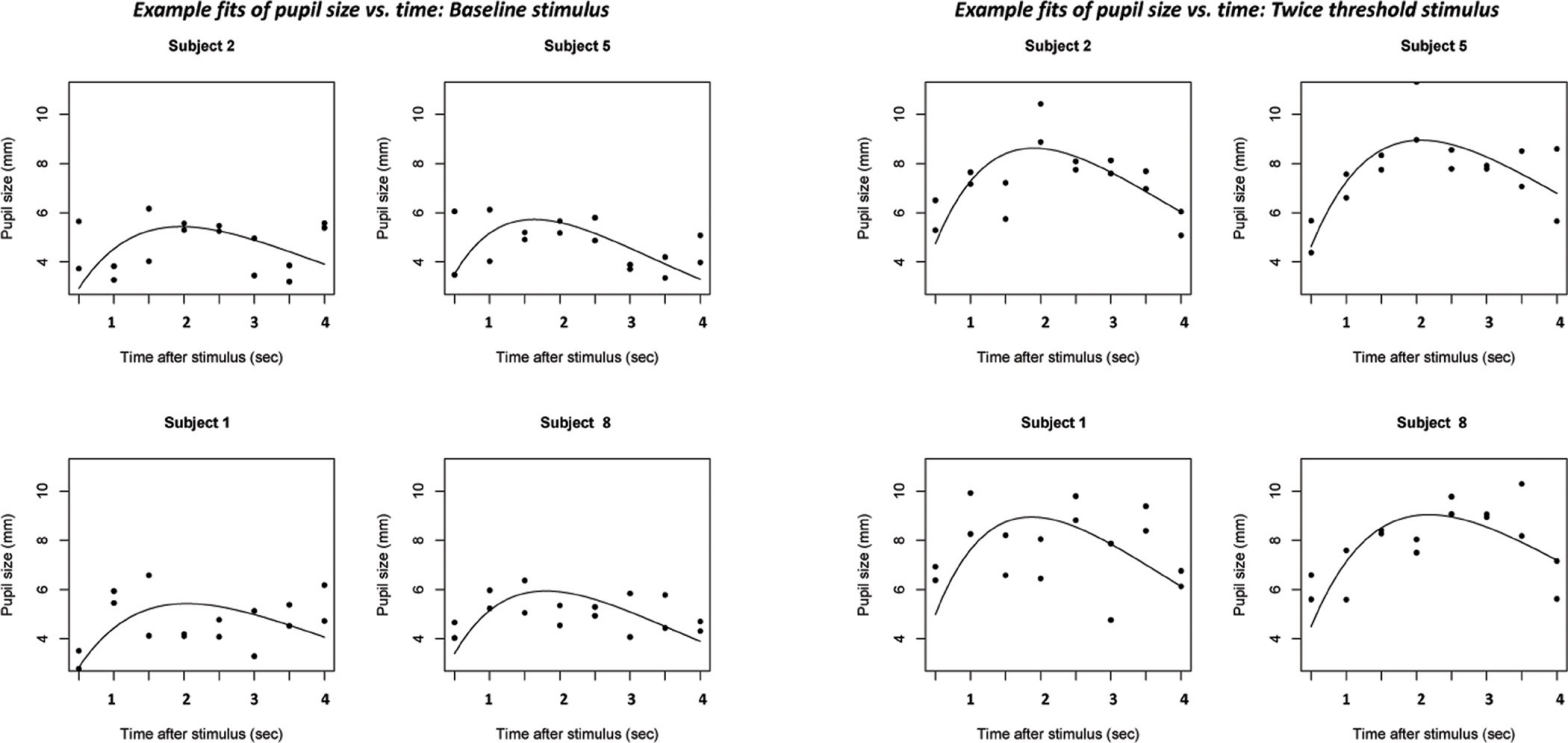
Example hierarchical fits of pupil size versus time for 4 (randomly selected) subjects. Left panel, 4 baseline stimuli and right panel same 4 subjects’ stimuli at twice threshold. Each time point has mechanical and chemical stimulus measurements.

**Fig 3:**
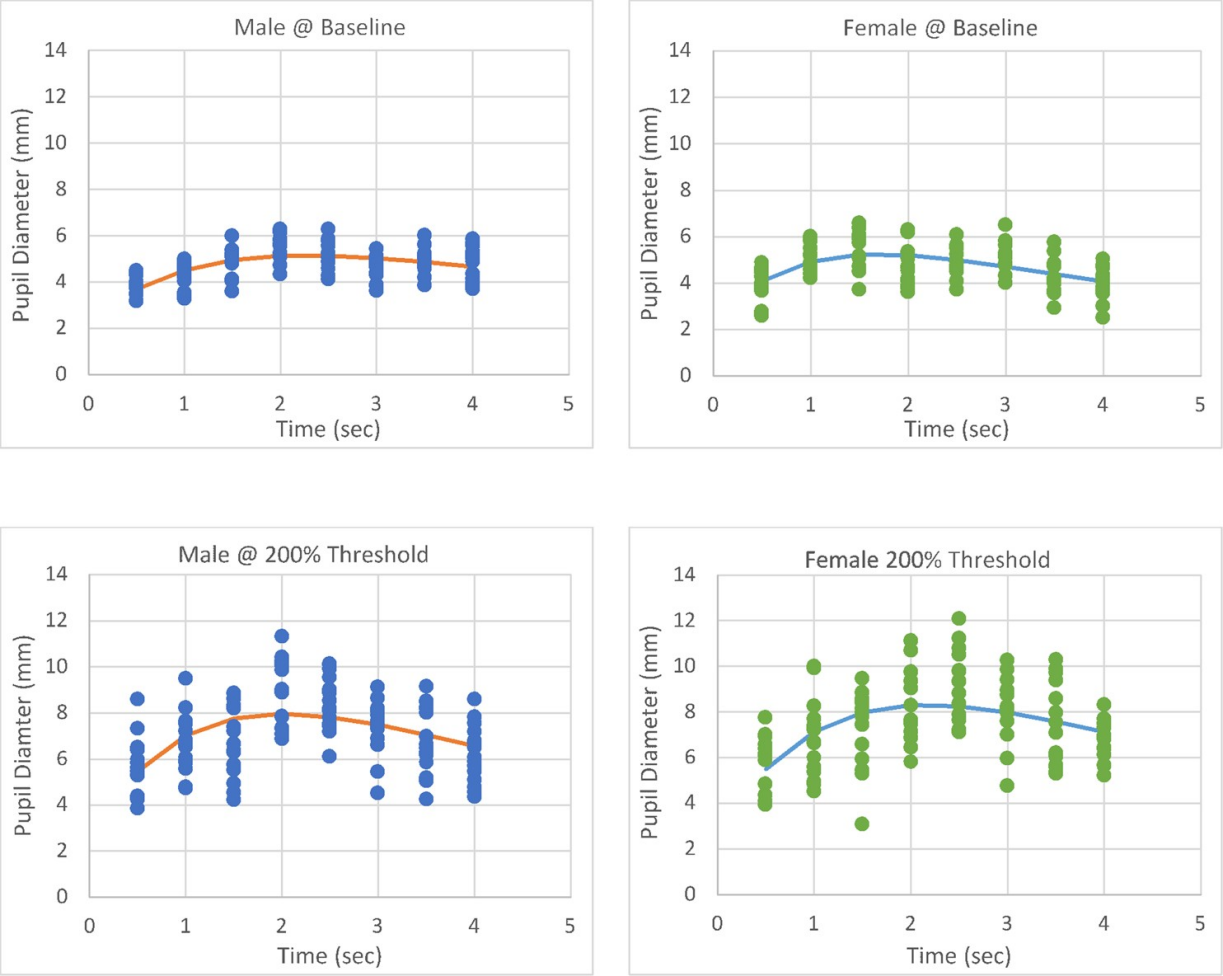
Group pre- and post-stimulus pupil diameter for mechanical corneal stimulation in male and female subjects.

**Fig 4:**
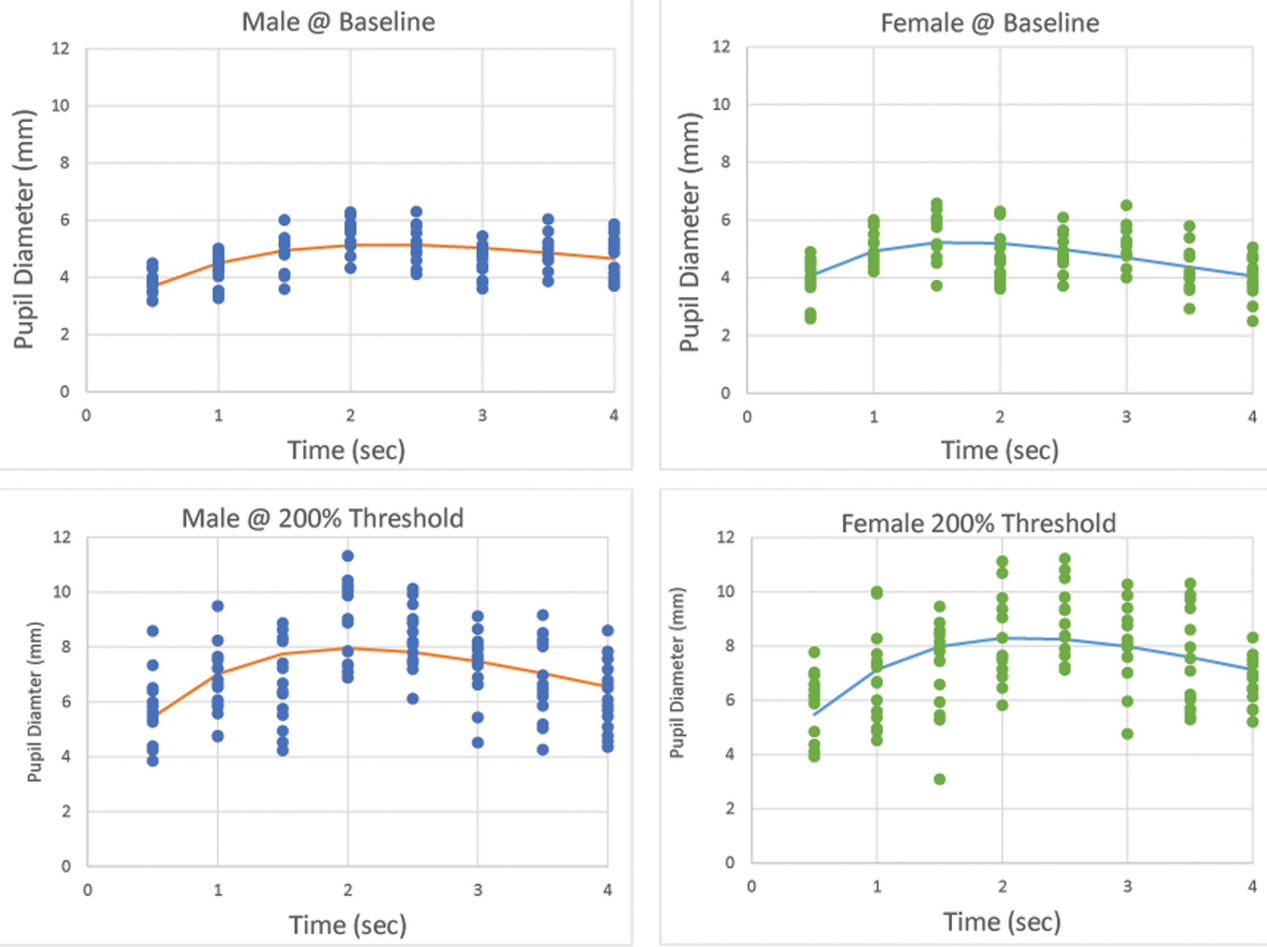
Group pre- and post-stimulus pupil diameter for chemical corneal stimulation in male and female subjects.

An example of the mixed model results on randomly selected participants for different stimulus intensities is in Fig 2. The left panel is using data from baseline stimulation and the right panel from twice-threshold intensities. Illustrating that the stimulus does have an effect on the pupil, the difference in the ‘size” component (B) was statistically significant comparing baseline to twice-threshold stimuli (paired t(_df = 29_) -41.702, p< 0.001). Figs 3 and 4 show the comparisons of these fits for male and female participants.

### Mean pupil diameters

**Fig 5:**
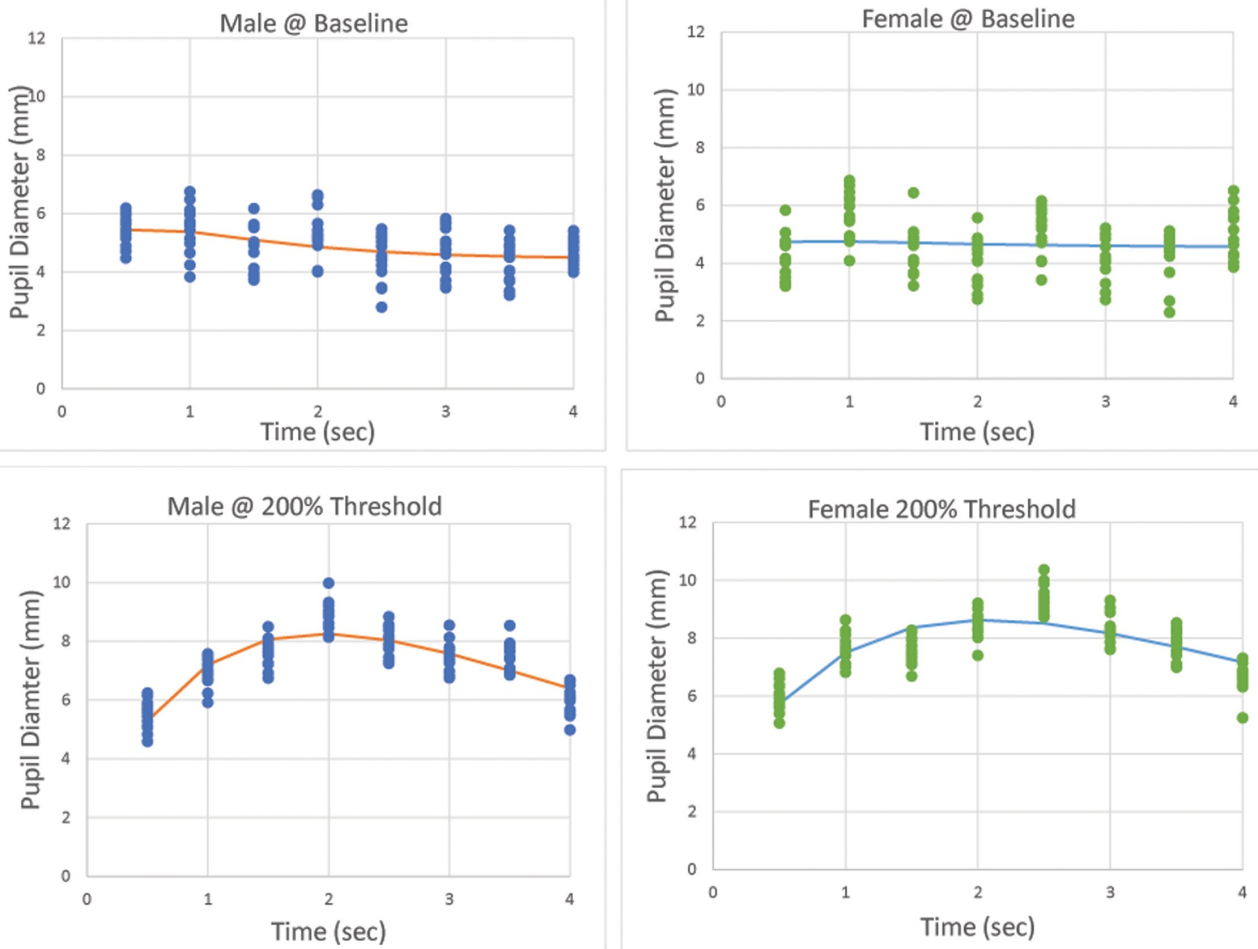
Mean pupil diameter across different stimulus intensities for mechanical (blue)/chemical (red) corneal stimulation experiments (upper panel) and for male (blue) and female (red) subjects (lower panel). Error bars denote 95% confidence interval.

In order to examine integrated (“area under curve”) performance we also looked at the average effects over the 8 second post-stimulus interval. A summary of the mean pupil diameters for males and females across the different stimulus intensities and modalities can be found in Table 1 below. Pupil diameters at baseline (before each measurement session), 0% threshold (the catch trials when the esthesiometer intensity setting was zero) and with mechanical and chemical stimulation of differing intensities stratified by modality are shown in Fig 5.

**Table 1:** Mean ±(SD) pupil size between males and females for corneal stimulation experiments.

		Sex	Mean Pupil Diameter (mm)	(±) Std. Deviation
Baseline	Mechanical Stimulus	Male	4.5	0.7
		Female	4.7	0.7
		Total	4.6	0.8
	Chemical Stimulus	Male	4.6	0.7
		Female	4.7	0.8
		Total	4.6	0.9
	Total	Male	4.5	0.7
		Female	4.7	0.8
		Total	4.6	0.8
0% Threshold	Mechanical Stimulus	Male	4.8	0.7
		Female	4.5	0.7
		Total	4.7	0.7
	Chemical Stimulus	Male	4.7	0.6
		Female	4.6	0.7
		Total	4.7	0.6
	Total	Male	4.8	0.6
		Female	4.6	0.7
		Total	4.7	0.7
Threshold	Mechanical Stimulus	Male	6.2	0.4
		Female	6.1	0.3
		Total	6.2	0.3
	Chemical Stimulus	Male	6.2	0.3
		Female	6.4	0.3
		Total	6.3	0.3
	Total	Male	6.2	0.3
		Female	6.3	0.4
		Total	6.3	0.3
150% Threshold	Mechanical Stimulus	Male	6.6	0.3
		Female	6.9	0.3
		Total	6.7	0.3
	Chemical Stimulus	Male	6.7	0.4
		Female	7.1	0.3
		Total	6.9	0.4
	Total	Male	6.6	0.3
		Female	7	0.3
		Total	6.8	0.4
200% Threshold	Mechanical Stimulus	Male	7.1	0.2
		Female	7.5	0.4
		Total	7.3	0.3
	Chemical Stimulus	Male	7.2	0.3
		Female	7.7	0.3
		Total	7.5	0.4

On average, pupil diameter increased from baseline as the corneal apical stimulus intensity increased. This happened regardless of whether mechanical or chemical stimulation occurred *(*ANOVA *F(4*,*224) = 356*.*6*, *p <* 0.05*)*. At 200% threshold, average pupil diameter was greater than at all other stimulus intensities (Tukey HSD, all *p <* 0.05).

### Effects of stimulus modality and stimulus intensity on pupil diameter

There was no difference in average pupil size between chemical and mechanical stimulation based on stimulus intensity *(*ANOVA *F(4*,*224) = 0*.*1*, *p >* 0.05*)*.

### Relationship between ipsi- and contralateral eye

**Fig 6:**
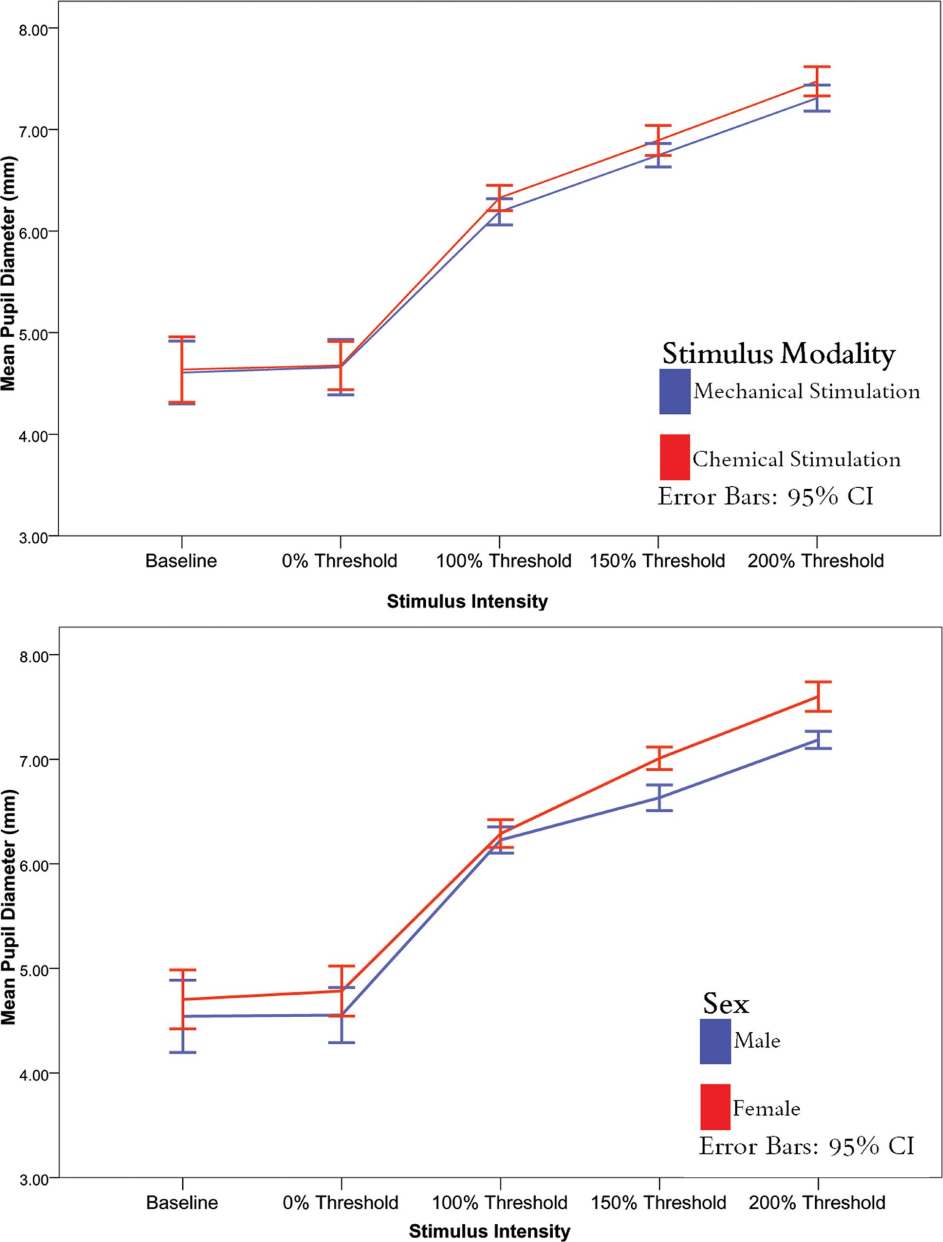
Box-plot of mean pupil diameter in the ipsilateral (stimulated) and the contralateral (unstimulated) eye after corneal mechanical (upper panel) and chemical (lower panel) stimulation. Error bars denote 95% confidence interval.

With mechanical and chemical stimulation of the cornea (Fig 6), there was no difference in pupil responses between the ipsilateral eye (stimulated eye [left eye]) and the contralateral (unstimulated) eye (paired t-test *t(14) = 0*.*6*, *and t(14) = 0*.*8*, mechanical and chemical respectively, both *p>0*.*05)*.

### Effects of sex and stimulus intensity on pupil diameter

There was a difference in pupil diameter between male and female subjects based on stimulus intensity *(*ANOVA *F(4*,*224) = 5*.*9*, *p <* 0.05*)*. Females had greater pupil diameters than males at 150% and 200% of threshold (Tukey HSD *p* < 0.05).

## Discussion

This is the first study to demonstrate the effect of systematic mechanical and chemical noxious ocular surface stimulation on pupil responses. Suprathreshold stimulation of the cornea appears to evoke a 2-to-3 second dose-response-like pupil diameter increase and a near-return to pre-stimulation diameter after 4 seconds.

The dilator and sphincter pupillae muscles of the iris are innervated by sympathetic and parasympathetic neurons respectively. Together, these smooth muscles work antagonistically to control pupil size [9]. The Edinger-Westphal nucleus, located in the midbrain, controls circular fibers within the sphincter pupillae to cause constriction of the pupils, mediating the pupillary light reflex[46]; it is however not involved in dilation. The hypothalamus controls radial muscles in the dilator pupillae to cause pupillary dilation [9]. The hypothalamus is also directly activated by ocular surface pain via the trigeminal pathway [28,29], therefore the pupil dilation response to nociceptive corneal stimuli observed in this study may support the idea that a neural connection exists within the hypothalamus linking dilation response and corneal nociception.

There have been reports of similar increase in pupil size in response to noxious stimulation. Chapman *et al*. [14] reported that pupil diameter increased when the intensity of painful fingertip stimulation was increased and proposed that this pupil size change was a complex defensive response to nociception mediated within the brain, and thus a good indicator of central processing of painful stimuli. Larson *et al*. [47] observed the effect of painful stimulation on physiological outcomes including pupil size, heart rate and arterial blood pressure in anesthetized subjects. They observed greater pupil sizes in subjects as putative painful stimulation increased and concluded that in comparison to heart rate and arterial blood flow, pupil responses provide greater sensitivity as a measurement of noxious stimulation. Oka *et al*. [15] studied the pupil dilation response to nociceptive stimuli and concluded that the increased pupil response to increasing noxious sensory input was not an artifact of cognitive effort, independent of painful experience, as some researchers had earlier suggested [48], but rather existed as part of a higher order defense response.

In our experiment reported here, we assessed the effect of the sex of subjects on the pupil dilation response to nociceptive corneal stimuli. From our results, there seems to be a difference in male and female pupil responses to threshold-scaled and therefore approximately equivalent noxious ocular surface stimulation: In females, pupil dilation was greater to suprathreshold stimuli than it was in males. Fillingim and Maixner [18] reviewed experiments conducted by others on gender differences in responses to noxious experimental stimuli using a ‘box-score’ or vote counting method and concluded that “females exhibit greater sensitivity to noxious stimulation than males”. Population based research by Unruh [19] shows that in comparison to men, there is a greater prevalence of back pain, arthritis and headaches among women. These findings may possibly be due to women having a tendency to honestly report pain (both acute and chronic) more often than men [49]. Hypotheses about sex differences include sex-role expectations [50], hormones [51,52], differences in skin thickness and body size [53] and sensory differences between men and women [54]. Ellermeier and Westphal [20] reported females had greater pupil dilation responses than males when high tonic pressure was applied to the fingers of subjects, and in that same study, females reported greater pain than males when the same amount of pressure was applied to the subjects’ fingers. Since it is not possible for one to voluntarily control his/her pupil response to noxious stimulation, the sex differences found in our study point to affective or sensory components of pain as opposed to subject bias in response [50] or attitudinal factors [55].

In this experiment, noxious stimulation of one eye caused pupil dilation in both eyes, much as withdrawal of light causes direct and consensual mydriasis. Similarly, Beradelli *et al*. [56] reported that ipsilateral electrical stimulation of the supraorbital branch of the trigeminal nerve caused a bilateral ocular reflex (blinking). The balance between the sympathetic and the parasympathetic system input determines the pupillary response as increased sympathetic innervation will cause a resultant pupillary dilation while decreased innervation will result in pupillary constriction. A possible explanation for the pupil dilation response may be linked to the fight or flight response–a physiological activation of the sympathetic nervous system that occurs when a harmful event (in this case noxious corneal stimulation) is perceived. The fight or flight response is characterized by the release of different hormones, and, for example, the adrenal medulla is known to secrete epinephrine and norepinephrine, the latter being the same neurotransmitter that modulates the iris dilator responsible for pupil dilation [57].

Situ and Simpson [38] reported comparable results to those of this study when they investigated the interaction between noxious corneal stimulation and tear secretion; mechanical and chemical corneal stimulation evoked increased reflex tearing. In addition, Situ and Simpson [38] reported that mechanical corneal stimulation produced the most reflex tearing. In a recent study by Alabi and Simpson [39], conjunctival redness increased in a dose dependent way to noxious corneal stimulation. The authors went on to further show that chemical stimulation of the cornea had a greater effect on conjunctival redness than mechanical corneal stimulation. However, in the current study, we have shown that the pupil response to corneal stimulation was the same regardless of whether a chemical or mechanical stimulus is applied. This leads to the speculation that the reflex arcs producing local changes in tear formation and conjunctival vessel dilation in response to painful corneal stimulation do not behave in the same way to that producing pupil dilation. The pupils respond to both mechanical and chemical stimulation in a similar manner therefore it can be assumed that the afferent parts of these reflex arcs read the information from mechanical and chemical nociceptors in an analogous way.

Increased tear secretion, conjunctival vessel dilation and more recently, the pupil dilation in response to corneal nociception shown in this study, result from different motor effects in different tissue. Because of this, and because each response to pain is monotonically related to pain (at least when scaled to detection thresholds), stimulating the cornea systematically, might be useful in assessing pain (and perhaps even consciousness), objectively and with greater accuracy by assessing multiple ocular responses. Treister *et al*. [58] suggested that a combination of several autonomic measures provided more accurate information than each single measures, and so a combination of these physiological responses to pain could provide better characterization of the pain process.

Our pupil dilations in response to painful suprathreshold corneal stimuli were comparable to some recent results reported in the literature although details are difficult to match since factors such as subjective stimulus intensity (that we did not measure) or fixation target luminance were not uniform across studies, so numerical comparisons are somewhat fuzzy. Nevertheless, there are similarities: For example, our pupil dilations were maximum approximately 2 seconds after stimulation and this is very similar to that reported by Hofle *et al*. [59], who used pressure pain on fingers as stimuli. In a different study, Hofle *et al*. [60] showed that the small pupil dilation effect occurred much sooner than ours when participants just viewed a painful stimulus compared to an innocuous one. On the other hand, our pupil dilation maxima occurred somewhat sooner than those reported by Eisenach *et al*. [61], perhaps not surprisingly, since they used thermal stimulation of the forearm or calf. The % maximum change was and % average change of pupil diameters in our study (see methods) were 79.7% and 57.4 respectively, generally quite high, compared to other reports, but again, similar concerns about different methods make direct comparisons problematic. For example, Oka *et al*. [62], and Hofle *et al*. [59], report pupil dilation in response to pain of less than 1 mm while we have average change approaching 3 mm. Scaled differently, Eisenach *et al*. [61], show around 15% change whereas our stimulation produced between 60%– 80% dilation. Although the timing and amount of dilation differ, these reports and ours show the same thing—dilation of the pupil after painful stimulation. On the other hand, the differences might reflect varying methods but also might point to the neural circuits driving the ocular response differing from those responsible for those driven by painful stimulation of the limbs.

Some experimental limitations of this study include a restriction to the stimulus intensity range, a limit to the amount of time selected to observe the pupil response and not observing several autonomic responses simultaneously. The highest stimulus intensity used was twice the threshold. The potential to go beyond twice threshold for both mechanical and chemical corneal stimulation would be beneficial because information regarding what happens to the pupil response at higher ocular surface stimulation intensities remains unknown. In assessing the pupil response to noxious stimulation, we limited the measurements to a time frame of 4 seconds. This method was chosen because prior studies involving painful stimulation and pupil responses [14,15,20] identified the greatest pupil response within the first three seconds of the post stimulus period; however, the potential to observe the pupil response over a longer period of time would provide important information regarding the complete nature of the pupillary response to noxious stimulation.

## Conclusion

In summary, this study provides some evidence that noxious mechanical and chemical stimulation of the central cornea evokes a dose dependent autonomic pupil dilation response. There seems to be a sex difference in the pupil dilation response, with women having a greater response than men when experiencing threshold-scaled equivalent amounts of noxious stimulation. This study serves as a basis for the characterization of the local stimulus-response neural circuitry relating nociceptive stimuli to autonomic responses and in combination with our work on completely separate autonomic circuits of bulbar conjunctival vessel dilation and reflex tearing suggests that the monotonic measurements of redness, tearing and pupils provide accurate, separable responses that reflect painful stimulus intensity.
